# Homocysteine as a potential indicator of endothelial dysfunction and cardiovascular risk in female patients with borderline personality disorder

**DOI:** 10.1186/s40479-021-00171-9

**Published:** 2022-01-03

**Authors:** Katharina Kern, Kathrin Sinningen, Luisa Engemann, Clara Maiß, Beatrice Hanusch, Andreas Mügge, Thomas Lücke, Martin Brüne

**Affiliations:** 1 Department of Psychiatry, Psychotherapy and Preventive Medicine, LWL University Hospital Bochum, Division of Social Neuropsychiatry and Evolutionary Medicine, Ruhr University Bochum, Alexandrinenstr. 1, 44791 Bochum, Germany; 2 University Hospital of Pediatrics and Adolescent Medicine, St. Josef-Hospital, Ruhr University Bochum, Bochum, Germany; 3 Bergmannsheil Bochum, Medical Clinic II, Department of Cardiology and Angiology, Ruhr University Bochum, Bochum, Germany

**Keywords:** Borderline personality disorder, Cardiovascular risk, Homocysteine, Childhood adversity, Chronic stress

## Abstract

**Background:**

There is increasing evidence suggesting that patients with Borderline Personality Disorder (BPD) are at greater risk of developing cardiovascular diseases (CVD) compared to the general population. Homocysteine (Hcy) has been discussed as a serum marker for endothelial dysfunction as a mechanism involved in CVD and has been shown to be associated with numerous psychiatric conditions. Pathophysiologically, there seems to be a link between Hcy and psychological stress mediated by abnormal activity of the autonomic nervous system. Accordingly, the present study sought to examine Hcy in BPD and to explore possible associations with clinical parameters.

**Methods:**

Plasma Hcy levels as well as conventional cardiovascular risk factors, such as blood pressure, BMI, smoking habits, HbA_1c_, HDL, LDL, and cholesterol, were examined in 49 young female in-patients diagnosed with BPD and 50 psychologically healthy control subjects matched for age and sex. Assessment of borderline symptom severity, childhood trauma, exposure to chronic stress, and quality of sleep was performed using self-reported questionnaires.

**Results:**

BPD patients showed significantly higher mean plasma Hcy concentrations compared to controls, though below ranges considered pathological. Moreover, Hcy correlated significantly with the severity of childhood trauma, chronic stress, and subjective sleep disturbances. In a regression model BPD diagnosis was found to predict Hcy levels best.

**Conclusion:**

In conclusion, young female BPD patients with no history of CVD show higher, though non-pathological, Hcy levels compared to healthy controls. Our findings seem to support the assumption that BPD is associated with increased risk of CVD, and that Hcy could serve as potential marker for risk evaluation of midlife CVD in BPD patients.

## Background

Borderline Personality Disorder (BPD) is a psychiatric disorder characterized by difficulties in emotion regulation, intense mood swings, impulsivity, unstable relationships, fragile self-perception, and self-injurious behavior, as well as chronic feelings of emptiness and fear of abandonment [1].

In recent years, there has been growing interest in the link between psychiatric conditions and physical health, most notably cardiovascular diseases (CVD) [2]. More specifically, a few studies have reported that patients with BPD are at higher cardiometabolic risk in midlife, whereby the experience of adversity in the form of early-life or chronic stress seems to play a role [3–5].

Indeed, a large number of studies have explored the impact of adverse childhood experiences, such as emotional neglect or abuse, to the development of borderline symptoms including emotion dysregulation and impulsivity [6–8]. Conversely, difficulties in emotion regulation contribute to the escalation of interpersonal distress, which creates a vicious circle with regard to maladaptive stress regulation [9, 10], which over time could increase the risk for CVD [11, 12]. Consequently, depression, anxiety, personality factors and character traits, such as hostility and anger, social isolation, and chronic life stress [13], all of which may accompany BPD, have been discovered to promote the development of coronary atherosclerosis.

Consistent with these considerations, hypertension and arteriosclerosis [14], stroke [15], and ischemic heart disease [5] occur more frequently in BPD than in the general population, whereby known risk factors for CVD including obesity [16], physical inactivity, tobacco smoking [17], metabolic syndromes [18], and poor quality of sleep [19] exert cumulative effects on the CVD risk in BPD.

Against this background, there is an essential need for identifying potential markers of heightened risk of CVD in people diagnosed with BPD, especially in light of the onset of the disorder in late adolescence or early adulthood, where preventive measures may be more effective than later in life. Homocysteine (Hcy) could be such a candidate for several reasons. Hcy is a non-proteinogenic amino acid and intermediate product of the methionine metabolism. Hcy has been considered as a potential correlate of an individual’s overall health status [20]. Moreover, elevated plasma levels of Hcy are discussed as a risk factor for vascular disease, independent of other risk factors such as hypercholesterolemia, hypertension, or cigarette smoking [21, 22]. Indeed, research has shown associations between elevated Hcy levels and coronary heart disease, cardiovascular as well as cerebrovascular disease [23]. Hcy essentially contributes to the progression of endothelial dysfunction, which is considered as a core factor in the development of atherosclerosis, and therefore increased risk of CVD [24, 25]. Furthermore, high Hcy plasma levels can potentiate adverse effects of other co-existing CVD risk factors, e.g. cigarette smoking and hypertension [26].

Aside from elevated Hcy plasma levels found in patients with homocystinuria, various vitamin deficiencies (vitamins B_6_, B_9_, and B_12_), renal failure, hypothyroidism, methylenetetrahydrofolate reductase (MTHFR) deficiency, and diabetes [27], Hcy plasma levels are also associated with physical inactivity, medication, such as anticonvulsant agents, tobacco use, male gender, and age [28].

Moreover, psychological distress, especially anger and hostility, seem to affect Hcy levels [29–31]. Although mechanisms underlying stress-induced increase of Hcy are not entirely understood, sympatho-vagal activation appears to play a predominant role [30]. Considering the significant presence of early life stress, chronic stress, as well as maladaptive stress regulation in BPD, it seems plausible to assume a link between BPD and Hcy levels, especially in light of the concept of “allostatic load”, that is, the accumulation of somatic consequences of an over-burdened stress-coping system [32]. Indeed, we recently found evidence for heightened allostatic load in an independent sample of young females with BPD [33].

With regard to psychiatric disorders, elevated Hcy has been reported in patients with depression, schizophrenia, obsessive-compulsive disorder, as well as posttraumatic stress disorder (PTSD) [34–39], while studies are lacking for BPD. Interestingly, a recent study indicates that shorter sleep duration correlates with higher Hcy, especially in women and obese individuals [40].

Taken together, there is a clear rationale for the study of CVD risk factors in BPD, including the necessity of a comprehensive risk assessment in young patients with BPD without a history of CVD as a means of primary prevention of CVD in this at-risk group. As Hcy is a direct correlate for endothelial dysfunction [24], and may increase the adverse effects of other CVD risk factors [26], we sought to examine Hcy in BPD and to explore its association with common risk factors for CVD as well as with childhood adversity, chronic stress, and quality of sleep. Specifically, we hypothesized that BPD patients would display higher levels of Hcy compared to controls, and that Hcy would correlate with the above-mentioned somatic, as well as stress-associated psychological factors.

## Material and methods

### Participants

In this cross-sectional clinical study 49 female in-patients diagnosed with BPD according to the Diagnostic and Statistical Manual of Mental Disorder Fifth Edition (DSM-5) [1] and a structured clinical interview [41] were enrolled at the Department of Psychiatry, Psychotherapy and Preventive Medicine, LWL University Hospital, Ruhr-University Bochum, Germany. All patients were seen by at least two independent experienced clinicians, as they were first diagnosed in the out-patient department, with verification of the diagnosis following admission to in-patient treatment. For comparison, 50 healthy female control participants (HC) were recruited via advertisement and screened for mental illnesses using the Mini-DIPS, a short version of the Diagnostic Inventory of Mental Disorders [42]. Exclusion criteria for all participants included a history of CVD, male sex, pregnancy, and, additionally, any past and current psychiatric conditions for the HC. Comorbid disorders and medication of patients with BPD are shown in Table 1.

**Table 1:** Comorbid conditions and medication of BPD group (*n* = 49) in absolute (*n*) and relative (%) quantity; patients had been abstinent from substance misuse for at least 6 weeks

	n	%
**Comorbidities**		
Recurrent depressive disorder	16	34.7
PTSD	13	26.5
Phobic/anxiety disorder	7	14.3
Substance misuse (alcohol, cannabinoids)	10	20.4
Eating disorder	7	14.3
ADHD	5	10.7
Hypothyroidism	1	2.0
**Medication**		
Without regular psychoactive medication	21	42.9
Antidepressant	27	55.1
Antipsychotic	14	28.6
Antidepressant + antipsychotic	13	26.5
Mood stabilizer/anticonvulsive	2	4.1
Other psychoactive medication	3	6.1

*PTSD* Posttraumatic Stress Disorder, *ADHD* Attention Deficit and Hyperactivity Disorder

The participants’ biological data are presented in Table 2 including systolic blood pressure (SBP), diastolic blood pressure (DBP), body mass index (BMI), waist-to-hip (WtH) ratio, and smoking habits. The latter was measured in pack years, i.e. the self-reported estimated number of years smoking multiplied by the number of packs of cigarettes (containing 20 cigarettes) smoked a day. Additionally, participants were classified as current smokers and never smokers (subjects who had never smoked on a regular basis). SBP and DBP were measured at rest in a sitting position using an automatic blood pressure monitor. In-patients received 30 Euros for taking part in the study, HC received 40 Euros, to reimburse additional travel expenses. Written informed consent was obtained from all participants before enrollment. This study was approved by the Ethics Committee of the Medical Faculty of the Ruhr-University Bochum, Germany, (Reference number: 18–6456-BR) and was carried out in full accordance with the Declaration of Helsinki.

**Table 2:** Biological data, serum markers, and Hcy of BPD and HC group with mean (SD), and Analyses of Variance

Dependent Variable	BPD	HC	F(1, 94)	η^**2**^	p
Age (years)	23.72 (4.6)	24.10 (3.9)	*.196*	*.002*	.659
Homocysteine (μmol/l)	2.99 (.58)	2.48 (.65)	*16.191*	*.147*	< .001
Pack years	3.61 (5.6)	.23 (1.1)	*17.350*	*.156*	< .001
BMI (kg/m^2^)	28.57 (7.4)	22.40 (3.0)	*29.258*	*.237*	< .001
Waist-to-hip ratio	.78 (.04)	.74 (.04)	*27.979*	*.229*	< .001
SBP (mmHg)	128.09 (12.5)	115.52 (9.5)	*31.160*	*.249*	< .001
DBP (mmHg)	83.04 (9.0)	75.76 (7.3)	*19.137*	*.169*	< .001
HDL (mg/dl)	56.28 (15.8)	71.68 (16.4)	*21.850*	*.189*	< .001
LDL (mg/dl)	99.93 (37.9)	102.70 (29.7)	*.160*	*.002*	.690
Cholesterol-HDL ratio	3.33 (1.26)	2.76 (.55)	*8.362*	*.082*	.005
HbA_1c_ (%)	5.18 (.30)	4.92 (.22)	*24.735*	*.208*	*<* .001
TSH (mIU/l)	1.47 (.69)	1.82 (.78)	*5.404*	*.054*	.022

*BMI* body mass index, *SBP* systolic blood pressure, *DBP* diastolic blood pressure

### Questionnaires

All study participants were asked to fill out various questionnaires in German. The short version of the Borderline Symptom List (BSL-23) was used to assess typical symptoms and symptom severity of BPD [43]. The short version of the Childhood Trauma Questionnaire (CTQ) comprising 28 self-rating items was used to measure subjective traumatic childhood experiences [44]. The CTQ has five subscales, i.e. emotional abuse, physical abuse, sexual abuse, emotional neglect, and physical neglect [45]. The level of maltreatment for each component was categorized as none or minimal, low to moderate, moderate to severe, and severe to extreme. Additionally, we calculated the CTQ global score. Exposure to chronic stress was assessed by the 57-item Trier Inventory for Chronic Stress (*Trierer Inventar zum Chronischen Stress*, TICS) [46]. Chronic stress was categorized as follows: work overload, social overload, pressure to perform, work discontent, excessive demands from work, lack of social recognition, social tensions, social isolation, and chronic worrying. These factors were again classified into two groups: high demand and lack of satisfaction. The questionnaire also yields a screening score for chronic stress (SSCS). Finally, participants were asked to complete the 19-item Pittsburgh Sleep Quality Index (PSQI) [47]. This questionnaire assesses subjective sleep quality and disturbances over a period of 1 month. With a total score of 0 to 21, whereby higher scores indicate worse quality of sleep, it evaluates the following seven components: sleep duration, subjective sleep quality, sleep latency, sleep efficiency, sleep disturbances, use of sleeping medication, and daytime dysfunction.

### Material/procedure

Venous blood sampling was performed using ethylenediaminetetraacetic acid (EDTA) and serum-gel monovettes (Sarstedt, Germany). Subsequently, the following parameters were analyzed in the central laboratory of the BG University Hospital Bergmannsheil Bochum immediately after sampling: glycated hemoglobin (HbA_1c_), total cholesterol, HDL-cholesterol, LDL-cholesterol, and thyroid-stimulating hormone (TSH). Conversely, Hcy was analyzed at the Immunological Laboratory of the University Hospital of Pediatrics and Adolescent Medicine, St. Josef-Hospital, Bochum, where the procedure has been established. Thus, within a time lag of 1 week to the first appointment, fasting venous blood samples were collected in EDTA monovettes (Sarstedt, Germany) and were promptly put on ice. Plasma was obtained by centrifugation (4000x g, 10 min, 4 °C) and stored at − 80 °C until further analysis of Hcy. Upon completion of the entire sample collection, an Enzyme-linked Immunosorbent Assay (ELISA) Kit (EKX-UAP3O8–96, Nordic BioSite, Sweden) was used to measure homocysteine levels in plasma in a total of three batches.

### Statistical methods

Statistical analysis was performed using IBM® SPSS® Statistics for Windows, version 26.0 (IBM Corp., Armonk, NY, USA). All tests were run two sided and results were considered significant with *p* < .05. A Shapiro-Wilk test was used to assess deviation from normality, which occurred in some variables. Group differences for biological data, Hcy, serum markers, and questionnaires were analyzed in a MANOVA. Post-hoc univariate ANOVAs were performed for every dependent variable. Correlations were determined using Spearman’s rho for variables based on pooled BPD and HC data. Bonferroni correction was applied to correct for multiple comparisons. A stepwise multilinear regression was calculated to determine which variables predicted Hcy values.

## Results

### Group comparisons

A MANOVA showed a statistically significant difference between the BPD and HC group on the combined dependent variables regarding biological data, serum markers, and Hcy (F (10, 83) = 10.994, *p* < .001, partial η^2^ = .614, Wilk’s Λ = .386). Post-hoc univariate ANOVAs were performed for every dependent variable. Results are shown in Table 2. BPD patients had significantly higher total plasma Hcy compared to HC (F (1, 94) = 16.191, *p* < .001, partial η^2^ = .147). Significant group differences were also found concerning smoking (pack years), BMI, WtH ratio, as well as SBP and DBP. In addition, significant differences between the groups occurred for HDL, cholesterol/HDL ratio, HbA_1c_ and TSH, but not LDL.

Thirty-eight of all 99 participants were current smokers (32 of whom belonged to the BPD group) and showed significantly higher Hcy levels than non-smokers (Mean = 2.88, SD = .67 vs. Mean = 2.60, SD = .65; F (1, 97) = 4.442, *p* = .038).

Accordingly, we performed a series of univariate ANOVAs with the most relevant cardiovascular risk factors that also correlated with Hcy levels as co-variates. These analyses showed that the group difference in Hcy remained significant when controlling for smoking, where the number of pack years had no significant impact on the dependent variable (i.e. Hcy); (F = 2.768, df = 1, *p* = .099). Similarly, neither SBP (F = 2.474, df = 1, *p* = .119) nor HbA_1_c (F = .853, df = 1, *p* = .358) had a significant impact on Hcy, such that group differences remained highly significant when controlling for these variables. All other potential CVD serum markers that differed between the groups (as shown in Table 2) had no impact on the group difference in Hcy.

A MANOVA showed statistically significant differences between the BPD and HC group on the combined dependent variables regarding the questionnaires, (F (12, 84) = 52.211, *p* < .001, partial η^2^ = .882, Wilk’s Λ = .118). Post-hoc univariate ANOVAs were performed for every dependent variable. Patients with BPD scored significantly higher on all four questionnaires, including subscales (Table 3). For each CTQ subscale the level of maltreatment in the HC group was classified as none or minimal. Mean scoring results of the BPD group revealed the following childhood maltreatment exposures: severe to extreme emotional abuse, low to moderate physical abuse, moderate to severe sexual abuse, moderate to severe emotional neglect, and moderate to severe physical neglect. BPD patients also showed more exposure to chronic stress as they scored about 16 points higher in the TICS SSCS, (F (1, 95) = 130.591, *p* < .001, η^2^ = .579). A PSQI global score higher than 5 indicates poor quality of sleep [47]. Accordingly, 93.6% (*n* = 44 of 47) of the BPD group reported poor quality of sleep, whereas this was the case in only 30.0% (*n* = 15 of 50) of the HC group. Similarly, patients with BPD had significantly higher PSQI global scores, which, on average, was 7.1 points higher than in the HC group (F (1, 95) = 141.013, *p* < .001, η^2^ = .597).

**Table 3:** Questionnaire results of BPD and healthy control group, mean (SD), and Analyses of Variance

Dependent Variable	BPD	HC	F(1, 95)	η^**2**^	p
BSL Sum Score	52.36 (19.0)	4.46 (3.7)	304.012	.762	< .001
CTQ Emotional Abuse	16.79 (5.8)	7.72 (3.0)	95.280	.501	< .001
CTQ Physical Abuse	8.55 (4.8)	5.84 (2.4)	12.678	.118	.001
CTQ Sexual Abuse	8.92 (6.0)	5.12 (.8)	19.798	.172	< .001
CTQ Emotional Neglect	16.38 (5.9)	7.16 (2.7)	101.134	.516	< .001
CTQ Physical Neglect	10.32 (3.5)	5.86 (1.5)	66.689	.412	< .001
CTQ Sum Score	60.94 (18.4)	32.70 (8.2)	97.103	.505	< .001
TICS SSCS	31.55 (7.0)	15.56 (6.8)	130.591	.579	< .001
TICS High Demand	45.32 (15.6)	35.96 (12.4)	10.718	.101	.001
TICS Lack of Satisfaction	66.70 (14.9)	30.36 (12.0)	175.391	.649	< .001
PSQI Global Score	11.61 (3.7)	4.48 (2.0)	141.013	.597	< .001
PSQI Subjective Sleep Quality	1.94 (.7)	.90 (0.5)	62.863	.398	< .001

*BSL* Borderline Symptom List, *CTQ* Childhood Trauma Questionnaire, *TICS* Trier Inventory for Chronic Stress;

*SSCS* Screening Scale for Chronic Stress, *PSQI* Pittsburgh Sleep Quality Index

### Correlations

When examining pooled BPD and HC data, Hcy correlated significantly with the number of pack years, blood pressure, HbA_1c_, as well as CTQ and PSQI scores with *p* < .05. After Bonferroni correction with *p* = .05/14 = .00357 (i.e. *p*-value corrected for the number of variables) only HbA_1c_, and the CTQ sum score still correlated significantly. In contrast, no significant correlations occurred between Hcy and age, BMI, WtH, HDL, TSH, or TICS SSCS. Table 4 shows Spearman’s correlations for additional relevant variables including Bonferroni correction.

**Table 4:** Spearman’s correlations (pooled BPD and HC data)

	Hcy	Age	BMI	WtH	Pack Years	SBP	DBP	HDL	LDL	HbA_1_c	TSH	BSL	CTQ	TICS SSCS
Age	−.002													
BMI	.033	.206												
WtH	.126	.172	.475^b^											
Pack Years	.248	.114	.272 ^a^	.371^b^										
SBP	.288 ^a^	.080	.354^b^	.329 ^b^	.303 ^b^									
DBP	.248	.231	.382 ^b^	.314 ^b^	.280 ^a^	.654 ^b^								
HDL	−.115	−.148	−.385 ^b^	−.447 ^b^	−.358 ^b^	−.208	−.105							
LDL	−.139	.261	.136	−.043	.023	.009	.184	−.064						
HbA_1c_	.280 ^b^	.264 ^b^	.384 ^b^	.429 ^b^	.257	.340 ^b^	.170	−.355 ^b^	.163					
TSH	−.104	.013	−.044	−.294 ^a^	−.276 ^a^	−.171	−.151	.043	.178	−.140				
BSL	.221	−.155	.465 ^b^	.416 ^b^	.527 ^b^	.416 ^b^	.305 ^b^	−.408 ^b^	−.090	.404 ^b^	−.326 ^b^			
CTQ	.302 ^b^	−.037	.462 ^b^	.361 ^b^	.573 ^b^	.383 ^b^	.287 ^a^	−.463 ^b^	−.077	.444 ^b^	−.181	.711 ^b^		
TICS SSCS	.197	−.071	.411 ^b^	.342 ^b^	.471 ^b^	.372 ^b^	.250	−.375 ^b^	−.086	.398 ^b^	−.272 ^a^	.870 ^b^	.678 ^b^	
PSQI	.212	−.148	.449 ^b^	.430 ^b^	.434 ^b^	.420 ^b^	.249	−.434 ^b^	−.070	.357 ^b^	−.144	.735 ^b^	.628 ^b^	.713 ^b^

*BMI* body mass index, *WtH* waist-to-hip ratio, *SBP* systolic blood pressure, *DBP* diastolic blood pressure, *BSL* Borderline Symptom List, sum score, *CTQ* Childhood Trauma Questionnaire, sum score, *TICS* Trier Inventory for Chronic Stress, *SSCS* Screening Scale for Chronic Stress, *PSQI* Pittsburgh Sleep Quality Index, global score

^a^ Correlation is significant at the 0.01 level (2-tailed)

^b^ Bonferroni-corrected significance *p* < 0.05/14 = .00357

Partial correlation analyses showed that correlations between Hcy and the CTQ sum score remained significant when controlling for current smoking (Spearman’s ρ = .223, *p* = .028), SBP (ρ = .201, *p* = .049), HbA_1_c (ρ = .222, *p* = .031), depression (ρ = .313, *p* = .002), and PTSD (ρ = .318, *p* = .001). Also, correlations between Hcy and CTQ subscales of emotional as well as physical neglect were significant when current smoking (ρ = .247, *p* = .015 and ρ = .204, *p* = .045, respectively), depression (ρ = .328, *p* = .001 and ρ = .276, *p* = .006), and PTSD (ρ = .323, *p* = .001 and ρ = .276, *p* = .006) was controlled for. No significant correlations were found between Hcy values and medication or comorbidities.

### Regression analysis

To examine which of the variables predicted Hcy best, we performed a stepwise multilinear regression analysis with Hcy as dependent variable, HbA_1c_ and CTQ sum score (both variables correlated significantly with Hcy even with Bonferroni-corrected significance *p* < .00357), BPD diagnosis, as well as depression and PTSD (as potentially confounding variables) as independent variables. An analysis of standard residuals was performed, which showed that the data contained no outliers. Also, data met the assumption of independent errors, did not display collinearity, and were approximately normally distributed. Only significant predictors were included in the stepwise regression model as variables were added based on their *p*-values (*p* < .05). In the first step, only BPD diagnosis was taken up into the model (B = .520, *b* = 389, *t* = 4.074, *p* < .001, Model 1). Specifically, diagnosis predicted roughly 14% of the variance of Hcy (F (1, 93) = 16.601, p < .001, R^2^ = .151, adjusted R^2^ = .142). In a second and final step depression was added to the model (Model 2). Model 2 with BPD diagnosis and depression as covariates predicted roughly 19% of the variance of Hcy (F (1, 92) = 11.68, p < .001, R^2^ = .203, adjusted R^2^ = .185). The variables HbA_1c_, CTQ sum score, and PTSD were excluded from the model.

## Discussion

The present study sought to explore possible associations of Hcy as an indicator for endothelial dysfunction and cardiovascular risk with childhood trauma, chronic stress, subjective quality of sleep, and biological markers of cardiovascular risk in female patients diagnosed with BPD. As predicted, plasma levels of Hcy were higher in patients, and group difference remained significant when controlling for smoking, systolic blood pressure, and HbA_1_c, which correlated with Hcy, but also differed between BPD patients and HC. However, in both groups Hcy levels were below ranges that are considered pathological. In a regression model BPD diagnosis appeared as the most significant predictor for Hcy levels. This result is compatible with previous studies reporting higher Hcy levels in patients with other psychiatric disorders, including PTSD [37, 48], obsessive-compulsive disorder [35], and depression [36], suggesting that increased Hcy is not specific to BPD.

Indeed, there are several known risk factors for CVD that correlate with Hcy, including smoking, blood pressure, and HbA_1c_ [28, 49, 50], which is in line with the findings of the present study. The mechanisms in which Hcy may be directly or indirectly involved comprise oxidative processes, smooth muscle cell proliferation, endothelial cell damage, platelet activation, thrombosis, and hypertension [11, 24, 51]. More particularly, Hcy may induce endothelial dysfunction mediated by accumulation of the endogenous NO synthase inhibitor asymmetric dimethylarginine (ADMA) [25]. Correspondingly, Kanzelmeyer et al. (2012) showed elevated ADMA concentrations in patients with hyperhomocysteinemia [52]. Moreover, Hcy impacts on the lipid and cholesterol metabolism and may also have pro-inflammatory properties [11]. With respect to tobacco use, 38 participants were current smokers and showed significantly higher Hcy levels than non-smokers. Furthermore, significant correlations between Hcy, SBP, and HbA_1c_ emerged, yet causal directions cannot be inferred from these findings. In any event, our research corroborates findings suggesting that BPD patients display a higher general CVD risk profile compared to a nonclinical comparison group, which may clinically manifest only in mid-age [3, 5]. Indeed, compared to HC, young female patients with BPD, who were mostly in their 20s or 30s, showed higher BMI, higher HbA_1c_, and higher SBP as well as DBP. These findings are also in line with the high rates of comorbid physical health conditions such as obesity [16], diabetes [17], and arterial hypertension [14] among patients with BPD. Unlike previous research [37, 53] we did not observe an association between age and Hcy, most likely because we deliberately chose to include young women with no history of CVD. It is also worth emphasizing that most measures were still within normal ranges, including Hcy, which could, however, be relevant for follow-ups over the life-span.

As in many other studies, patients with BPD also reported higher exposure to childhood trauma, chronic stress, as well as poorer quality of sleep [6, 8–10, 54, 55]. Interestingly, we found that Hcy correlated with the severity of childhood trauma, chronic stress, and inversely with subjective quality of sleep, whereas it did not correlate with BMI. Partial correlations revealed that the correlation between Hcy and childhood trauma remained significant when controlling for smoking, HbA_1_c, depression and PTSD. In addition, childhood trauma, chronic stress, and BMI correlated with poor quality of sleep. In a general vein, this could suggest that the exposure to childhood trauma and current psychosocial stress is associated with poor sleep and sleep deprivation, which in turn may activate the stress system [56]. As Kashani et al. (2012) hypothesized, poor quality of sleep may be the link between maladaptive stress coping and risk of CVD [57]. In line with previous studies in BPD reporting more objective sleep fragmentation, less sleep efficiency, as well as poor subjective sleep quality [19] and concomitant daytime consequences [58], we found that BPD patients had more pronounced subjective sleep disturbances compared to controls. With regard to BMI, Vorona et al. (2005) reported that obesity has an adverse impact on sleep quality [59], which could explain the high number of poor sleepers in the BPD group. This seems to be compatible with a study in a nonclinical sample, in whom shorter sleep duration correlated with higher Hcy serum levels, especially in obese individuals [15].

The association of Hcy with childhood adversity and chronic stress also deserves more attention. Indeed, childhood trauma may cause a chronic activation of the hypothalamic-pituitary-adrenal axis, which may have serious consequences on health later in life [60]. For example, physical and sexual child abuse are predictors of early adulthood CVD [61] and are associated with coronary heart disease [62]. As early life stress may also pave the way for greater exposure to chronic psychosocial stress later in life, these effects are difficult to disentangle. In any event, several studies have demonstrated that acute and chronic stress can increase the Hcy level [29–31], and promote endothelial dysfunction [13, 63]. Black and Garbutt (2002), for example, have proposed that repetitive episodes of chronic stress can cumulatively lead to clinically relevant atherosclerosis, attributed, in part, to stress-induced elevated Hcy levels and endothelial dysfunction, suggesting the need for more research of the role of Hcy in the context of chronic stress [11].

The present study has several limitations. As we exclusively examined young females with BPD, the study results cannot be generalized for males, especially since Hcy values show considerable sex-dependent differences [64]. Secondly, folate, vitamin B_12_, and protein consumption were not measured, which could have an impact on Hcy plasma levels. Thirdly, the potential influence of exercise on Hcy was not controlled for, which is an omission because it is known that physical activity can lower Hcy [28]. Fourthly, quality of sleep, as well as smoking habits, were measured by self-report, which admittedly are fraught by the risk of exaggeration or denigration. It would therefore be desirable to include objective (e.g., polysomnographic) data in future studies. Fifthly, despite significantly higher Hcy levels in BPD patients compared to HC, all levels ranged below concentrations considered pathological. Thus, replication in independent samples, including comparisons with other psychiatric diagnostic groups is warranted. For this young age group, Hcy may be seen as a potential indicator rather than a direct risk factor for CVD risk later in life. However, as this is solely correlative research our findings do not allow any conclusions regarding causality. Finally, several BPD patients were diagnosed with comorbid PTSD and/or depression; however, the numbers were too small to analyze subgroups, which would be worth considering in future studies. Moreover, future studies should include a hospital control group instead of a healthy control group to reduce selection bias.

## Conclusion

In summary, to the best of our knowledge, this is the first study that has examined the complex interplay of CVD risk, childhood trauma, chronic stress, and quality of sleep in a group of somatically healthy individuals with BPD. Young female BPD patients displayed higher Hcy levels compared to healthy controls, whereby the clinical significance of this finding is, to some degree, unclear and requires replication. However, our results substantiate the assumption that BPD is associated with a greater risk for poor somatic health. A more speculative conclusion is that Hcy may serve as a potential indicator for monitoring cardiovascular health in this patient group. Finally, our findings may raise a number of interesting questions for future research, including the potential effect of psychotherapy on preventing somatic disease.

## Abbreviations

ADMA: Asymmetric dimethylarginine
BMI: Body mass index
BPD: Borderline Personality Disorder
BSL: Borderline Symptom List
CTQ: Childhood Trauma Questionnaire
CVD: Cardiovascular disease
DBP: Diastolic blood pressure
EDTA: Ethylenediaminetetraacetic acid
HC: Healthy controls
HbA_1c_: Glycated hemoglobin
Hcy: Homocysteine
HDL: High-density lipoprotein cholesterol
LDL: Low-density lipoprotein cholesterol
MTHFR: Methylenetetrahydrofolate reductase
PSQI: Pittsburgh Sleep Quality Index
PTSD: Post-Traumatic Stress Disorder
SBP: Systolic blood pressure
SSCS: Screening Scale for Chronic Stress
TICS: Trier Inventory for Chronic Stress
TSH: Thyroid-stimulating hormone
WtH: Waist to hip

## References

[1] American Psychiatric Association (2013). Diagnostic and statistical manual of mental disorders, fifth edition (DSM-5®).

[2] Levine GN, Cohen BE, Commodore-Mensah Y, Fleury J, Huffman JC, Khalid U (2021). Psychological health, well-being, and the mind-heart-body connection: a scientific statement from the American Heart Association. Circulation..

[3] Barber T, Ringwald WR, Wright AG, Manuck SN (2019). Borderline personality disorder traits associate with midlife cardiometabolic risk: Center for Open Science.

[4] Dong M, Giles WH, Felitti VJ, Dube SR, Williams JE, Chapman DP, Anda RF (2004). Insights into causal pathways for ischemic heart disease: adverse childhood experiences study. Circulation..

[5] Moran P, Stewart R, Brugha T, Bebbington P, Bhugra D, Jenkins R, Coid JW (2007). Personality disorder and cardiovascular disease: results from a national household survey. J Clin Psychiatry.

[6] Bierer LM, Yehuda R, Schmeidler J, Mitropoulou V, New AS, Silverman JM, Siever LJ (2003). Abuse and neglect in childhood: relationship to personality disorder diagnoses. CNS spectr.

[7] Lieb K, Zanarini MC, Schmahl C, Linehan MM, Bohus M (2004). Borderline personality disorder. Lancet.

[8] Paris J, Zweig-Frank H, Guzder J (1994). Psychological risk factors for borderline personality disorder in female patients. Compr Psychiatry.

[9] Pagano ME, Skodol AE, Stout RL, Shea MT, Yen S, Grilo CM (2004). Stressful life events as predictors of functioning: findings from the collaborative longitudinal personality disorders study. Acta Psychiatr Scand.

[10] Scott LN, Levy KN, Granger DA (2013). Biobehavioral reactivity to social evaluative stress in women with borderline personality disorder. Personal Disord.

[11] Black PH, Garbutt LD (2002). Stress, inflammation and cardiovascular disease. J Psychosom Res.

[12] Steptoe A, Kivimäki M (2012). Stress and cardiovascular disease. Nat Rev Cardiol.

[13] Rozanski A, Blumenthal JA, Kaplan J (1999). Impact of psychological factors on the pathogenesis of cardiovascular disease and implications for therapy. Circulation..

[14] El-Gabalawy R, Katz LY, Sareen J (2010). Comorbidity and associated severity of borderline personality disorder and physical health conditions in a nationally representative sample. Psychosom Med.

[15] Chen M-H, Hsu J-W, Bai Y-M, Su T-P, Li C-T, Lin W-C (2017). Risk of stroke among patients with borderline personality disorder: a nationwide longitudinal study. J Affect Disord.

[16] Powers AD, Oltmanns TF (2013). Borderline personality pathology and chronic health problems in later adulthood: the mediating role of obesity. Personal Disord..

[17] Keuroghlian AS, Frankenburg FR, Zanarini MC (2013). The relationship of chronic medical illnesses, poor health-related lifestyle choices, and health care utilization to recovery status in borderline patients over a decade of prospective follow-up. J Psychiatr Res.

[18] Kahl KG, Greggersen W, Schweiger U, Cordes J, Correll CU, Frieling H (2013). Prevalence of the metabolic syndrome in patients with borderline personality disorder: results from a cross-sectional study. Eur Arch Psychiatry Clin Neurosci.

[19] Winsper C, Tang NKY, Marwaha S, Lereya ST, Gibbs M, Thompson A, Singh SP (2017). The sleep phenotype of borderline personality disorder: a systematic review and meta-analysis. Neurosci Biobehav Rev.

[20] Cleophas TJ, Hornstra N, van Hoogstraten B, van der Meulen J (2000). Homocysteine, a risk factor for coronary artery disease or not? A meta-analysis. Am J Cardiol.

[21] Clarke R, Daly L, Robinson K, Naughten E, Cahalane S, Fowler B, Graham I (1991). Hyperhomocysteinemia: an independent risk factor for vascular disease. N Engl J Med.

[22] Homocysteine Studies Collaboration (2002). Homocysteine and risk of ischemic heart disease and stroke: a meta-analysis. JAMA..

[23] Bautista LE, Arenas IA, Peñuela A, Martinez LX (2002). Total plasma homocysteine level and risk of cardiovascular disease. J Clin Epidemiol.

[24] Lai WKC, Kan MY (2015). Homocysteine-induced endothelial dysfunction. ANM..

[25] Stühlinger MC, Oka RK, Graf EE, Schmölzer I, Upson BM, Kapoor O, et al. Endothelial dysfunction induced by hyperhomocyst(e)inemia: role of asymmetric dimethylarginine. Circulation. 2003:933–8. 10.1161/01.CIR.0000085067.55901.89. 10.1161/01.CIR.0000085067.55901.89 12912818

[26] Graham IM, Daly LE, Refsum HM, Robinson K, Brattström LE, Ueland PM (1997). Plasma homocysteine as a risk factor for vascular disease. Eur Concerted Action Project JAMA.

[27] Refsum H, Smith AD, Ueland PM, Nexo E, Clarke R, McPartlin J (2004). Facts and recommendations about total homocysteine determinations: an expert opinion. Clin Chem.

[28] Eikelboom JW, Lonn E, Genest J, Hankey G, Yusuf S (1999). Homocyst(e) ine and cardiovascular disease: a critical review of the epidemiologic evidence. Ann Intern Med.

[29] Sawai A, Ohshige K, Kura N, Tochikubo O (2008). Influence of mental stress on the plasma homocysteine level and blood pressure change in young men. Clin Exp Hypertens.

[30] Stoney CM (1999). Plasma homocysteine levels increase in women during psychological stress. Life Sci.

[31] Stoney CM, Engebretson TO (2000). Plasma homocysteine concentrations are positively associated with hostility and anger. Life Sci.

[32] McEwen B (2000). Allostasis and allostatic load implications for Neuropsychopharmacology. Neuropsychopharmacol..

[33] Otto B, Kokkelink L, Brüne M. Borderline personality disorder in a “life history theory” perspective: evidence for a fast “pace-of-life-syndrome”. Front Psychol. 2021. 10.3389/fpsyg.2021.715153. 10.3389/fpsyg.2021.715153 PMC8350476 34381406

[34] Atmaca M, Tezcan E, Kuloglu M, Kirtas O, Ustundag B (2005). Serum folate and homocysteine levels in patients with obsessive-compulsive disorder. Psychiatry Clin Neurosci.

[35] Balandeh E, Karimian M, Behjati M, Mohammadi AH. Serum vitamins and homocysteine levels in obsessive-compulsive disorder: a systematic review and Meta-analysis. NPS. 2021:1–14. 10.1159/000514075. 10.1159/000514075 33744893

[36] Bottiglieri T, Laundy M, Crellin R, Toone BK, Carney MW, Reynolds EH (2000). Homocysteine, folate, methylation, and monoamine metabolism in depression. J Neurol Neurosurg Psychiatry.

[37] Jendricko T, Vidović A, Grubisić-Ilić M, Romić Z, Kovacić Z, Kozarić-Kovacić D (2009). Homocysteine and serum lipids concentration in male war veterans with posttraumatic stress disorder. Prog Neuro-Psychopharmacol Biol Psychiatry.

[38] Levine J, Stahl Z, Sela BA, Gavendo S, Ruderman V, Belmaker RH (2002). Elevated homocysteine levels in young male patients with schizophrenia. Am J Psychiatry.

[39] Tiemeier H, van Tuijl HR, Hofman A, Meijer J, Kiliaan AJ, Breteler MMB (2002). Vitamin B12, folate, and homocysteine in depression: the Rotterdam study. Am J Psychiatry.

[40] Chen T-Y, Winkelman JW, Mao W-C, Yeh C-B, Huang S-Y, Kao T-W (2019). Short sleep duration is associated with increased serum homocysteine: insights from a National Survey. J Clin Sleep Med.

[41] Wittchen H-U, Zaudig M, Fydrich T (1997). SKID. Strukturiertes klinisches Interview für DSM-IV. Achse I und II. Handanweisung.

[42] Margraf J. Mini-DIPS. Berlin, Heidelberg: Springer Berlin Heidelberg; 1994.

[43] Bohus M, Kleindienst N, Limberger MF, Stieglitz R-D, Domsalla M, Chapman AL (2009). The short version of the borderline symptom list (BSL-23): development and initial data on psychometric properties. PSP..

[44] Klinitzke G, Romppel M, Häuser W, Brähler E, Glaesmer H (2012). Die deutsche version des childhood trauma questionnaire (CTQ) - psychometrische Eigenschaften in einer bevölkerungsrepräsentativen Stichprobe. [the German version of the childhood trauma questionnaire (CTQ): psychometric characteristics in a representative sample of the general population]. Psychother Psychosom Med Psychol.

[45] Bernstein DP, Stein JA, Newcomb MD, Walker E, Pogge D, Ahluvalia T (2003). Development and validation of a brief screening version of the childhood trauma questionnaire. Child Abuse Negl.

[46] Schulz P, Schlotz W (1999). Trierer Inventar zur Erfassung von chronischem Streß (TICS): Skalenkonstruktion, teststatistische Überprüfung und Validierung der Skala Arbeitsüberlastung. Diagnostica..

[47] Buysse DJ, Reynolds CF, Monk TH, Berman SR, Kupfer DJ (1989). The Pittsburgh sleep quality index: a new instrument for psychiatric practice and research. Psychiatry Res.

[48] Levine J, Timinsky I, Vishne T, Dwolatzky T, Roitman S, Kaplan Z (2008). Elevated serum homocysteine levels in male patients with PTSD. Depression Anxiety.

[49] Haj Mouhamed D, Ezzaher A, Neffati F, Douki W, Najjar MF (2011). Effect of cigarette smoking on plasma homocysteine concentrations. Clin Chem Lab Med.

[50] Lim U, Cassano PA (2002). Homocysteine and blood pressure in the third National Health and nutrition examination survey, 1988-1994. Am J Epidemiol.

[51] Esse R, Barroso M, Tavares de Almeida I, Castro R (2019). The Contribution of Homocysteine Metabolism Disruption to Endothelial Dysfunction: State-of-the-Art. Int J Mol Sci.

[52] Kanzelmeyer N, Tsikas D, Chobanyan-Jürgens K, Beckmann B, Vaske B, Illsinger S (2012). Asymmetric dimethylarginine in children with homocystinuria or phenylketonuria. Amino Acids.

[53] Xu R, Huang F, Wang Y, Liu Q, Lv Y, Zhang Q (2020). Gender- and age-related differences in homocysteine concentration: a cross-sectional study of the general population of China. Sci Rep.

[54] Torgersen S (2009). The nature (and nurture) of personality disorders. Scand J Psychol.

[55] Zanarini MC, Williams AA, Lewis RE, Reich RB, Vera SC, Marino MF (1997). Reported pathological childhood experiences associated with the development of borderline personality disorder. AJP..

[56] Joo EY, Yoon CW, Koo DL, Kim D, Hong SB (2012). Adverse effects of 24 hours of sleep deprivation on cognition and stress hormones. J Clin Neurol.

[57] Kashani M, Eliasson A, Vernalis M (2012). Perceived stress correlates with disturbed sleep: a link connecting stress and cardiovascular disease. Stress..

[58] Selby EA (2013). Chronic sleep disturbances and borderline personality disorder symptoms. J Consult Clin Psychol.

[59] Vorona RD, Winn MP, Babineau TW, Eng BP, Feldman HR, Ware JC (2005). Overweight and obese patients in a primary care population report less sleep than patients with a normal body mass index. Arch Intern Med.

[60] Teicher MH, Andersen SL, Polcari A, Anderson CM, Navalta CP, Kim DM (2003). The neurobiological consequences of early stress and childhood maltreatment. Neurosci Biobehav Rev.

[61] Rich-Edwards JW, Mason S, Rexrode K, Spiegelman D, Hibert E, Kawachi I (2012). Physical and sexual abuse in childhood as predictors of early-onset cardiovascular events in women. Circulation..

[62] Rooks C, Veledar E, Goldberg J, Votaw J, Shah A, Bremner JD, Vaccarino V (2015). Long-term consequences of early trauma on coronary heart disease: role of familial factors. J Trauma Stress.

[63] Kershaw KN, Lane-Cordova AD, Carnethon MR, Tindle HA, Liu K (2017). Chronic stress and endothelial dysfunction: the multi-ethnic study of atherosclerosis (MESA). Am J Hypertens.

[64] Adeli K, Higgins V, Nieuwesteeg M, Raizman JE, Chen Y, Wong SL, Blais D (2015). Complex reference values for endocrine and special chemistry biomarkers across pediatric, adult, and geriatric ages: establishment of robust pediatric and adult reference intervals on the basis of the Canadian health measures survey. Clin Chem.

